# KIT-Associated Familial GIST Syndrome: Response to Tyrosine Kinase Inhibitors and Implications for Risk Management

**DOI:** 10.1093/oncolo/oyac120

**Published:** 2022-07-06

**Authors:** Alexandra Brodey, Valentinos Kounnis, Lara Hawkes, Robin L Jones, Terri P McVeigh, Elena Cojocaru

**Affiliations:** Department of Oncology, Oxford University Hospitals NHS Foundation Trust, Oxford, UK; Department of Oncology, Oxford University Hospitals NHS Foundation Trust, Oxford, UK; Oxford Centre for Genomic Medicine, Oxford University Hospitals NHS Foundation Trust, Oxford, UK; Sarcoma Unit, The Royal Marsden NHS Foundation Trust, London, UK; Institute of Cancer Research, London, UK; Institute of Cancer Research, London, UK; Cancer Genetics Unit, The Royal Marsden NHS Foundation Trust, London, UK; Department of Oncology, Oxford University Hospitals NHS Foundation Trust, Oxford, UK; Cancer Genetics Unit, The Royal Marsden NHS Foundation Trust, London, UK

## Abstract

Sporadic gastrointestinal stromal tumors (GIST) are rare tumors, with a median age at diagnosis of 60 years. Familial GISTs are very rare and typically associated with earlier onset, with an average age at diagnosis of 48 years. To date, just over 50 familial cases associated with a germline variant *KIT* or *PDGFRa* genes have been published. Therefore, there are many challenges in managing these patients, including the timing of starting systemic treatment, considering that most patients have been asymptomatic for a long period before being diagnosed, as well as the choice of tyrosine kinase inhibitor and the plan for surveillance.

It is uncertain if early diagnosis through screening of asymptomatic individuals improves overall survival. Screening could start from the age of 18 years but may be considered at earlier ages depending on the underlying genotype and family history. The long-term benefit of early diagnosis or palliative/prophylactic treatment with tyrosine kinase inhibitors is unknown as there are no data available. Long-term side effects of treatment with imatinib are rare but well documented and could be damaging in patients who have no or minimal disease.

We present the case of a 53-year-old Caucasian patient who was diagnosed with multifocal GIST and subsequently found to be a carrier of a pathogenic germline *KIT* variant in exon 11. We discuss the implication of treatment and genetic testing in this case and in familial KIT associated GISTs.

## Teaser

Familial gastrointestinal stromal tumors (GIST) are rare and associated with earlier onset. This article reports on the management of familial GIST and highlights the importance of access to molecular testing and genetic screening in asymptomatic or young patients.

**Key Points**

- Familial gastrointestinal stromal tumors (GIST) is a very rare occurrence and possibly underdiagnosed; patients often present with multifocal tumors and can be asymptomatic for a long time before diagnosis.
- Medical treatment should follow the guidelines for sporadic GIST according to molecular subtype of each individual. High-risk tumors are usually treated with tyrosine kinase inhibitors and surgery of the symptomatic lesions. Low- and moderate-risk tumors can be managed with surgery of the symptomatic lesions or surveillance.
- Germline testing should be sought in young patients and those with a family history of GIST.
- Testing to all family members should be offered when a germline *KIT* mutation has been identified in an individual.

## Patient History

**Figure 1.**
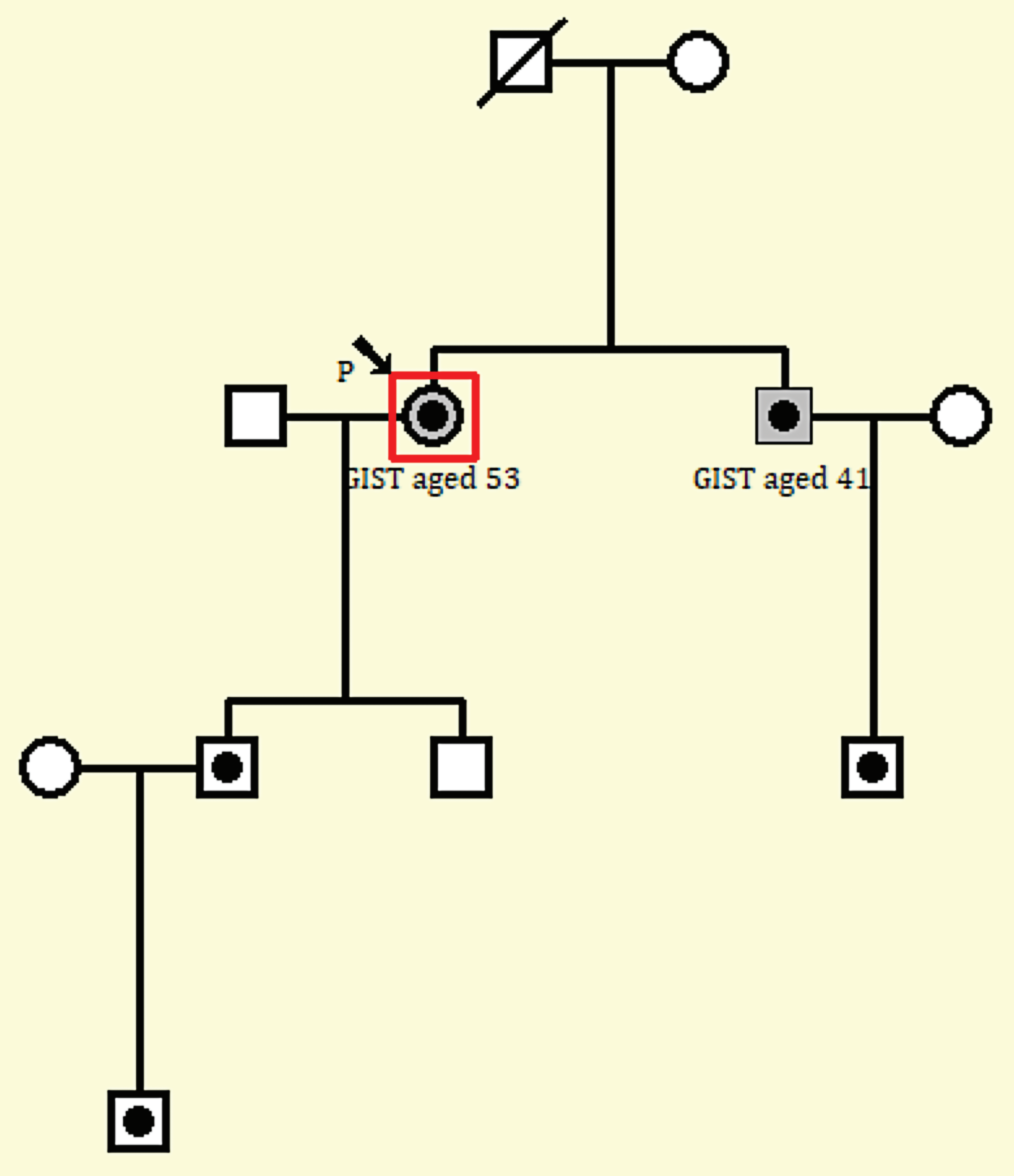
Pedigree. The individuals with framed black dots represent *KIT* germline carriers.

We present the case of a 53-year-old Caucasian female who was found to be a carrier of a pathogenic germline *KIT* variant in exon 11 (p.Asp579del) after predictive testing for a familial detected variant. This *KIT* exon 11 germline alteration was initially detected in her nephew, after he presented with unusual progressive hyperpigmentation after his father (our patient’s brother) developed a gastrointestinal stromal tumors (GIST) in his 40s (Fig. 1).^1^ At the time of testing, examination showed only mild freckles on her arms, with no rashes and no gastrointestinal symptoms.

After local multi-disciplinary meeting (MDT), she underwent a baseline abdominal magnetic resonance imaging (MRI) and esophagogastroduodenoscopy, based on international practice in management of similar families.^2^ The surveillance MRI scan, PET CT and biopsy, confirmed the presence of multifocal GISTs within the stomach, esophagus, pelvis, and small bowel. The histopathology confirmed a GIST, and tumor cells were positive for DOG-1, CD117, and CD34 on immunohistochemistry. No necrosis or mitotic figures were seen. Further contrast-enhanced CT thorax and abdomen demonstrated enlarged nodes around the stomach, mild splenomegaly, and a heterogeneous appearance to the bones with numerous tiny sclerotic lesions.

Our patient was commenced on systemic treatment with imatinib 400 mg once daily. Given the suspicion of systemic mastocytosis (SM) secondary to bone infiltration and splenomegaly, she was reviewed by a hematologist; however, such diagnosis was not confirmed due to low level of the blood tryptase (25.6 μg/L), although this test was done after commencing therapy with imatinib and therefore response to imatinib could not be excluded.

On the 3- and 6-month response assessment scan after commencing therapy with imatinib, the GISTs have considerably diminished in both size and FDG avidity, confirming an excellent response to therapy, which was maintained at 12 months with minimal side effects. Our patient is planned to undergo surgical removal of all the residual lesions which are amenable to surgery. The post-operative treatment will consist of imatinib for possibly life-long duration, depending on tolerance. It is possible that any SM symptoms will remain controlled by imatinib, and the patient will remain under joint care of the oncologist and hematologist.

Her affected brother also remains alive and well, having undergone surgical resection without systemic treatment. Testing of her family members has been initiated and the patient’s son (aged 30) and grandson (aged 6) have been found to carry the familial *KIT* exon 11 variant.

## Molecular Tumor Board

### Background on Familial KIT-Related GISTs

Gastrointestinal stromal tumors (GIST) are rare cancers with a median age at diagnosis of 60 years and equal distribution between males and females. They account for 0.1%-3% of all gastrointestinal neoplasms.^3^ Gastrointestinal stromal tumors can arise in various organs but most commonly are found in the stomach and small intestine, and often are diagnosed incidentally.^4,5^ Up to 85% of GIST have gain-of-function variation in *KIT* or *PDGFR*a genes.^4^ The remaining 15% of GISTs are associated with other genetic alterations, including in *NF1*, *SDH*, or *BRAF* genes.^6^

Treatment with imatinib, a tyrosine kinase inhibitor, in GIST harboring a variant in exon 11 of *KIT*, has significantly improved the survival for patients with advanced disease from 18 to more than 70 months.^7,8^ Response to imatinib is dependent on the specific underlying genotype, such as mutations in *KIT* exon 11 rendering a better response rate compared with *KIT* exon 9 variants.^5^ Avapritinib, a new targeted therapy, has proven excellent efficacy in *PDGFRα* p.Asp842Val GIST, known to be resistant to imatinib.^8,9^

Most *KIT/PDGFRa* variants in GIST are somatic in origin, but rare cases caused by constitutional variants in these genes have been reported. Fornasarig et al conducted a meta-analysis and identified 51 familial cases associated with a germline variant in either *KIT* (*n* = 45) or *PDGFRa* (*n* = 6).^10^ Familial GISTs are typically associated with earlier onset, with an average age at diagnosis of 48 years, compared with an average age of 60 years in sporadic cases.^11,12^ These tumors present frequently as multifocal disease and can affect the whole gastrointestinal tract.^10,13^ Some familial GISTs can present with indolent disease and a low risk of progression.^2,14,15^

In addition to GISTs, families harboring germline *KIT* variants often demonstrate variable clinical phenotypes caused by mast-cell activation (mastocytosis). Mastocytosis can be cutaneous, affecting mainly children and causing urticaria pigmentosa, characterized by small tan-red macules found on the upper and lower extremities and on the thorax and abdomen. In other cases, mast cell disorder can cause systemic symptoms, secondary to organ infiltration of mast cells. Individuals affected by SM can present with hepatosplenomegaly, anemia, dysphagia as well as anxiety or depression and can vary in severity, from indolent disease with slow progression to mast cell leukaemia.^16^ The management of mastocytosis depends on the severity of symptoms, including topical corticosteroids for treatment of mild-to-moderate cutaneous mastocytosis or antihistamines for generalized symptoms. In patients with advanced mastocystosis, tyrosine kinase inhibitors are used depending on the underlying genetic mutation (*KIT* or *PDGFRA*).^17^

Other cancers have been reported in carriers of germline *KIT* (g*KIT*) variants, although whether these cancers are attributable to the underlying genotype is unclear.^10^ Furthermore, a higher incidence of second malignancy has also been reported in patients with sporadic GISTs compared with the general population.^18,19^

Other familial GIST syndromes can be caused by constitutional variants in *NF1* or *SDH* genes, which seem to cause a more indolent disease. Often, *NF1-* or *SDH-*related GISTs are resistant to systemic therapy and their management include surgery of symptomatic lesions or surveillance, depending on the burden of their disease.^11,15^ Commonly, these familial GISTs will present with multi-focal lesions, some of causing serious complications, such as bleeding or bowel obstruction, necessitating immediate surgery.^20^ In other cases, a watch and wait approach is preferred and interventions only when progression of lesions is noticed or symptoms occur.^15^

### Efficacy of Tyrosine Kinase Inhibitors in Familial KIT-Associated GIST

Few case reports published to date have documented the response to TKI in familial GISTs caused by germline *KIT* variants (Table 1). Tarn et al identified a young patient who developed a GIST at the age of 37 and was found to carry the same germline pathogenic variant as our patient, *KIT* exon 11 (p.Asp579del).^21^ In this case, the patient’s tumor had a high mitotic index and was classified as high risk but had a clinical indolent disease which remained stable on imatinib.^21^

**Table 1.** Response to TKI in familial GISTs.

*KIT* variant	Exon	Age at diagnosis of GIST	Response to imatinib
c.1669T>A, (p.Trp557Arg)	11	Case 1: 36 Case 2: 23	Case 1 PFS 12 years after firs initiation Imatinib. Recurrence 1 year after stopping with PFS 15 months after re-initiating Imatinib(22) Case 2: No evidence disease at 15 months after Imatinib(22)
c.1733_1735delATG, (p.Asp579del)	11	40	PFS 9 months on imatinib (14)
c.1733_1735delATG, (p.Asp579del)	11	36F	Stable disease on imatinib post resection—no long term data (21)
c.1733_1735delATG, (p.Asp579del)	11	53F	Ongoing partial response at last FU (12 months on imatinib) *(our patient)*
c.1924A>G (p.Lys642Glu)	13	Case 1: 28M Case 2: M, unknown age	Case 1: died of the disease progression after imatinib was stopped for toxicity. No data on PFS (2) Case 2: stable after 8 years of treatment with imatinib (2)

In a case report published by Farag et al, another 52-year-old patient with a germline variant in *KIT* exon 11, p.Trp557Arg, with multiple GISTs was treated with imatinib and had a rapid tumor regression with subsequent disease stability for 7 years, followed by resection of residual disease.^22^ Two of the 3 patient’s children undergoing predictive testing were found to carry the familial variant. During their MRI screening, one of her daughters was diagnosed with multiple GISTs, and underwent resection followed by adjuvant imatinib for a duration of 3 years, after which an extension of the therapy was to be discussed.^22^

Another case report investigated the response to tyrosine kinase inhibitors in familial GISTs caused by a novel germline variant in *KIT* exon 13 p.Lys642Glu.^2^ Most of the tumors in the 20 affected patients from 3 large families included in this report were of low or intermediate risk of recurrent disease. Only 2 patients from this series received imatinib for metastatic disease and the best response was stable disease.^2^

In only one published case, treatment beyond imatinib has been described in familial GIST; this case relates to a patient with familial GIST due to a germline *KIT* exon 13 variant p.Asn655Lys.^10^ This patient received sunitinib as second line of treatment and remained stable for a total of 15 months, which is consistent with response rates in sporadic GIST cases.^10^

### Discussion and Recommendations for Management of Familial KIT-Related GIST

Only around 50 cases of KIT-associated familial GIST have been reported to date.^9,14,23^ However, with increasing availability of germline testing, this number is likely to increase. In the UK, NHS Genomic Services has expanded the criteria for germline testing of *KIT* and other GIST predisposition genes to patients affected before age 50 years, if there is associated mastocytosis, a family history of GIST or associated cancers.^23^

Guidelines for type and frequency of surveillance of affected individuals and unaffected carriers of germline pathogenic *KIT* variants are lacking. Some authors suggested that these patients should undergo regular medical checks, including yearly physical examination, regular abdominal ultrasound, MRI or CT/PET-CT or frequent esophagogastric endoscopies.^2,10,22^ Bachet et al have recommended a follow-up schedule with 2-3 yearly CT or MRI as a screening modality in unaffected carriers or in affected patients with small and asymptomatic GISTs; with intervals shortened to yearly for those patients with larger or symptomatic GISTs after an initial scan at 6 months.^2^ Farag et al proposed use of biannual MRI for at—risk carriers.^22^ MRI scans as a screening tool may be considered as an alternative to CT/PET-CT for those patients undergoing long-term surveillance to minimize long-term exposure to radiation.

Even if early diagnosis is facilitated, *KIT*-related GIST is often multi-focal, precluding an R0 surgical resection. Furthermore, some tumors occurring in individuals with hereditary predisposition to GIST can be indolent and could potentially remain asymptomatic depending on the mitotic and necrotic index and the burden of the disease. It is therefore uncertain if early diagnosis through screening of asymptomatic individuals improves overall survival. Furthermore, the age at which screening should commence in unaffected individuals is uncertain, given that the median age at diagnosis of GIST in gKIT carriers is 48, but considering that GISTs have been reported in adolescent/young adult carriers.^24,25^ Many authors propose starting screening from the age of 18 years but may be considered at earlier ages depending on the underlying genotype and family history.^2^

Whilst these screening procedures might be expensive and time consuming, the long-term benefit is also unknown, considering that, even after a resection with curative intent of a small number of GIST deposits, other GISTs will likely form throughout the individuals’ lifetime. A long-term preventive treatment with TKI in patients bearing a sensitive variant has been proposed by Bachet and colleagues; however, no long-term toxicity data are reported.^2^ Preclinical data on a murine model have demonstrated potential long-term effects on pregnancy and implantation, therefore preventative treatment with TKI in young patients must be very carefully considered.^26^ Whether long-term treatment with TKI or frequent imaging can increase further the risk of a second malignancy in this population remains to be determined.

We have very scattered information on the effect of other TKIs in familial GIST, but it is possible that many patients have not been identified as being part of a familial syndrome and therefore have been treated with sunitinib and regorafenib as per national guidelines.^10^ Therefore, unless there is a known resistant variant in the family, the first choice of TKI should be imatinib followed by sunitinib and regorafenib, as recommended by the national and international guidelines in the treatment of sporadic GISTs. Newer agents, such as avapritinib and ripretinib, might be of interest in case of resistance to the standard of care.^8^

Carriers of germline pathogenic *KIT* variants may also benefit from consultation with a hematologist before initiation of any systemic treatment with tyrosine kinase, to determine whether there are any signs of mast cell disease.

The age at which germline *KIT* testing should be considered is uncertain. Generally, genetic testing for variants associated with adult-onset cancer predisposition is deferred until individuals are mature enough to provide informed consent, usually as adults. However, germline testing may be considered at younger ages where clinical features associated with a condition may present in adolescence or even in childhood. Most authors advocate for earlier testing if preventative or treatment strategies are available that would modify the disease course or outcome.^27^ The non-neoplastic features associated with germline pathogenic variants in *KIT* may be evident early in childhood, such that testing in children is not unreasonable.

Carriers of pathogenic germline variants may avail several reproductive options to minimize their risk of passing the familial variant to their progeny. This may include pre-implantation genetic diagnosis, with implantation of only those embryos that do not carry the familial variant, or prenatal testing with termination of carrier embryos.

**Figure 2.**
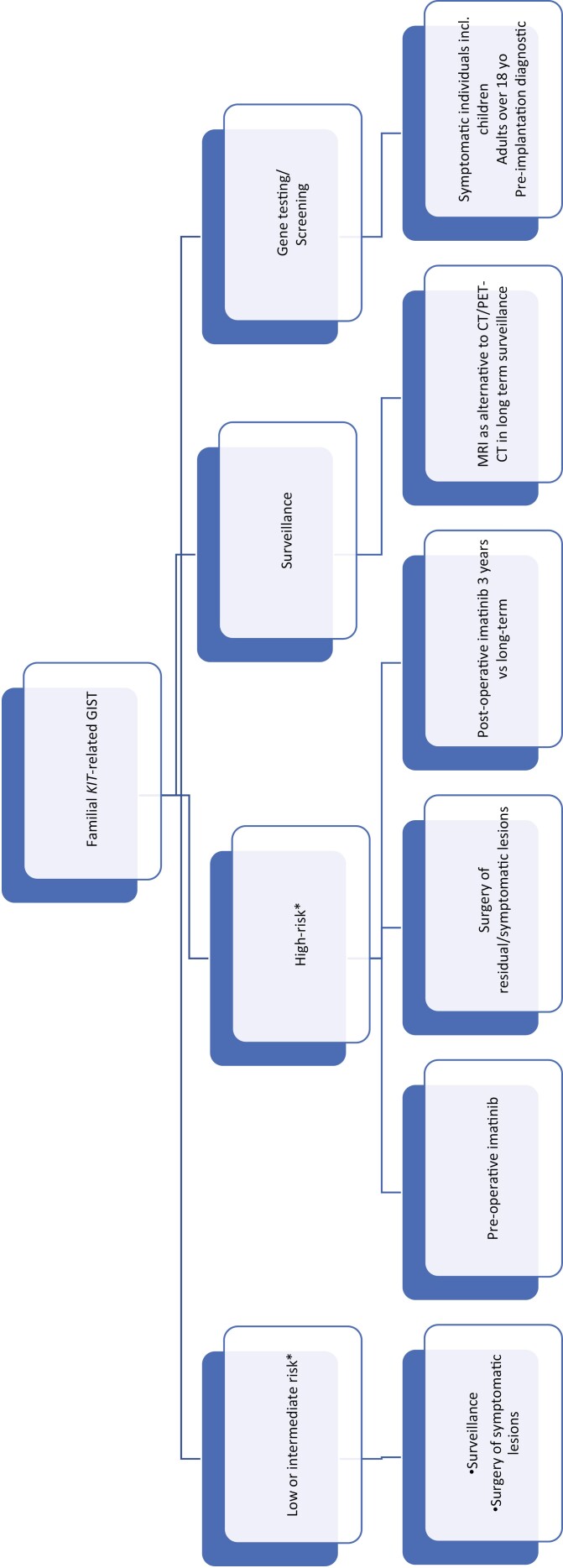
Proposed management algorithm for familial *KIT*-related GIST. ^*^As per modified NIH risk classification.^28^

We propose a management algorithm based on risk-stratification for familial *KIT-*exon 11 related GISTs, illustrated in Fig. 2.

### Patient Update

Our patient underwent surgery in March 2022 having completed 16 months of neo-adjuvant Imatinib. She underwent a laparotomy which found evidence of several gastric GISTs encompassing the proximal stomach and further peritoneal and caecal deposits were noted. She underwent resection of the small bowel GIST (maximum diameter 7 cm) and a peritoneal biopsy. Histopathology confirmed the small bowel excision as a GIST; however, the peritoneal biopsy showed fibrofatty tissue only.

## Conclusions

Familial *KIT*-associated GIST is rare and may be overlooked. Clinicians should consider germline testing in individuals affected at young ages or those with a family history of GIST and be particularly alert to non-neoplastic phenotypic features that suggest hereditary predisposition to GIST. It is likely that more carriers of germline pathogenic *KIT* variants will be identified as germline genetic testing becomes increasingly available. While some familial GISTs might behave indolent, the majority will likely necessitate local or systemic treatment with a tyrosine kinase inhibitor, and, in the absence of dedicated guidelines for *KIT*-associated Familial GISTs, the treatment guidelines for sporadic GIST harbouring somatic *KIT* variants can be followed. Long-term follow-up of affected germline *KIT* variant carriers is required to determine whether outcomes in such individuals differ compared with individuals affected with sporadic *KIT*-driven GISTs. Until formal guidelines are available, screening in asymptomatic carriers of germline KIT variants should be individualised and guided by underlying genotype and family history.

## Data Availability

The data underlying this article will be shared on reasonable request to the corresponding author.
